# Dual Immune Checkpoint Blockade in Synchronous Hepatocellular and Renal Cell Carcinoma: A Real-World Case Report of Durable Dual Response

**DOI:** 10.32604/or.2026.078258

**Published:** 2026-06-16

**Authors:** Piera Federico, Maria Anna Canciello, Barbara Granata, Davide Bosso, Bruno Daniele

**Affiliations:** 1 Medical Oncology Unit, Ospedale del Mare, Naples, Italy; 2 School of Specialization in Medical Oncology, Department of Human Pathology “G. Barresi”, University of Messina, Messina, Italy

**Keywords:** Renal cell carcinoma, hepatocellular carcinoma, dual checkpoint blockade, synchronous immunogenic tumors, case report

## Abstract

**Background:** The synchronous occurrence of hepatocellular carcinoma (HCC) and clear cell renal cell carcinoma (ccRCC) is rare and poses significant therapeutic challenges, particularly in elderly patients with comorbidities. Although both malignancies may respond to immune checkpoint inhibitors (ICIs), evidence supporting a unified immunotherapeutic approach remains limited. This report aims to describe the clinical course and outcomes of dual immune checkpoint blockade (ICB) in a patient with synchronous HCC and ccRCC. **Case Description:** We describe a patient in their 80s with synchronous advanced HCC and ccRCC, with underlying cirrhosis related to hepatitis C virus infection and cardiovascular comorbidities. Following multidisciplinary evaluation, treatment with nivolumab plus ipilimumab was initiated. Early radiologic assessment demonstrated a partial response in the renal lesion and an apparent increase in the hepatic lesion, consistent with pseudoprogression. Continued therapy led to progressive reduction of the hepatic lesion and sustained control of the renal tumor. At approximately 18 months of follow-up, both lesions showed durable radiologic response with excellent tolerability and no immune-related adverse events. At the time of writing (April 2026), the patient remains on treatment. **Conclusions:** This case highlights the feasibility and potential efficacy of a unified immunotherapeutic strategy in synchronous immunogenic malignancies. It also underscores the importance of careful interpretation of radiologic findings during immunotherapy, particularly in HCC where atypical response patterns such as pseudoprogression may occur. These findings provide real-world insight into personalized immunotherapy approaches for complex oncologic scenarios.

## 1 Introduction

Renal cell carcinoma (RCC) and Hepatocellular carcinoma (HCC) are two malignancies with distinct etiologies, molecular profiles, and anatomical origins, but both have shown responsiveness to immune checkpoint inhibition. Over the last decade, immunotherapy has become a cornerstone in the management of advanced RCC, with the combination of nivolumab (anti-PD-1) and ipilimumab (anti-CTLA-4) establishing a new standard of care in intermediate- and poor-risk patients, based on the CheckMate-214 trial [1]. Similarly, in HCC, the tumor’s immunogenic potential has prompted the development of several immune-based strategies, initially led by the CheckMate-040 study, which demonstrated durable responses with nivolumab and ipilimumab in the second-line setting [2].

Most recently, the phase III CheckMate-9DW trial has evaluated this same dual checkpoint blockade as a first-line approach in patients with advanced HCC, comparing it to the standard tyrosine kinase inhibitors sorafenib and lenvatinib. Preliminary results indicate encouraging survival outcomes, adding further support for this strategy in selected patients with well-preserved liver function [3]. While current first-line HCC therapy relies on atezolizumab–bevacizumab or durvalumab–tremelimumab combinations, dual PD-1/CTLA-4 blockade remains of interest, particularly in settings where systemic treatment is needed for multiple immune-responsive tumors.

The synchronous occurrence of advanced HCC and RCC is exceptionally rare and presents unique clinical challenges. There is a lack of standardized therapeutic guidance in this setting, and no consensus exists regarding whether to treat each tumor individually or to pursue a unified systemic approach. Here, we report the case of a patient in their 80s diagnosed with concurrent advanced HCC and biopsy-confirmed clear cell RCC, treated with combined nivolumab and ipilimumab. A durable dual tumor response with excellent tolerance was achieved. This case highlights the feasibility and potential effectiveness of a single ICB strategy in managing synchronous malignancies with overlapping immunologic features.

Written informed consent for publication was obtained from the patient. According to institutional policy of Ospedale del Mare, Naples, Italy, formal ethics committee approval is not required for single case reports; therefore, no ethical approval number is applicable.

This case report was prepared in accordance with the CARE guidelines, and the CARE checklist is provided as Supplementary Material S1.

## 2 Case History

A patient in their 80s was evaluated and treated at the Medical Oncology Unit, Ospedale del Mare, Naples, Italy. The patient had a medical history of hypertension, pharmacologically controlled with antihypertensive therapy, type 2 diabetes mellitus, osteoporosis, and previously eradicated hepatitis C virus (HCV) infection (HCV-RNA negative as of June 2024). Two isolated, self-limited episodes of atrial fibrillation were reported in the months preceding treatment initiation, with no need for specific ongoing therapy. No other cardiovascular comorbidities were documented, and cardiovascular status remained clinically stable during treatment. She presented in mid-2024 with right-sided abdominal pain. Her surgical history included hysterectomy with bilateral adnexectomy and cholecystectomy. No relevant family history of malignancy was reported.

Initial imaging with abdominal ultrasound and contrast-enhanced CT revealed a heterogeneous hepatic lesion in segment VIII (maximum diameter 43 mm) and a right mesorenal mass (40 mm), both suspicious for malignancy. MRI confirmed a hypervascular liver lesion in segment VIII (53 × 44 mm) with arterial phase enhancement and pseudocapsule formation, suggestive of HCC, and an enhancing solid renal mass (45 × 40 mm) consistent with renal cell carcinoma (Fig. 1A–F).

**Figure 1:**
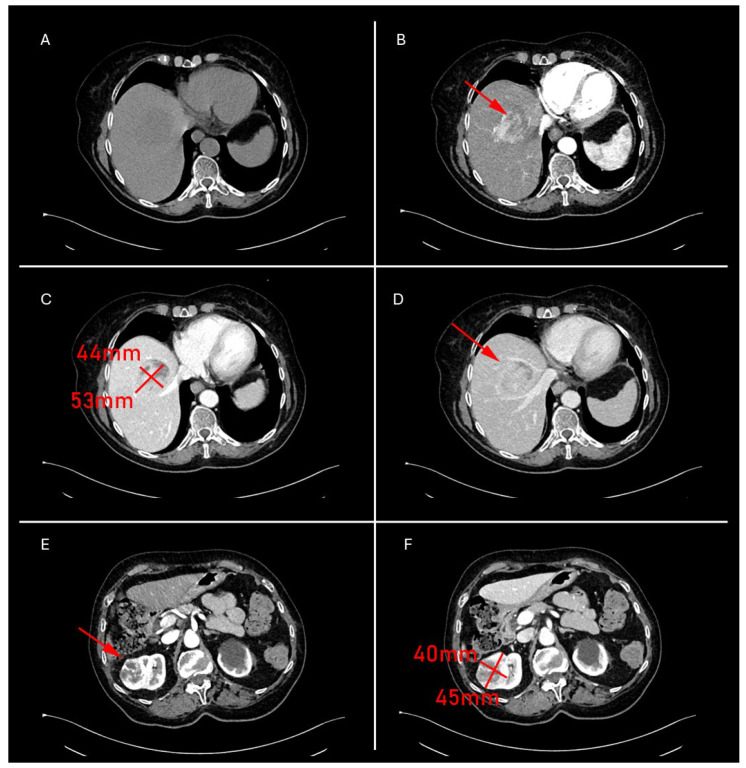
Baseline CT scan of the patient. (**A**–**D**) Hepatic lesion (HCC): (**A**) non-contrast phase; (**B**) arterial phase showing arterial hyperenhancement (red arrow); (**C**) venous phase showing lesion size (53 × 44 mm); (**D**) delayed phase demonstrating pseudocapsule appearance (red arrow). (**E**,**F**) Renal lesion (RCC): (**E**) arterial phase showing enhancing renal mass (red arrow); (**F**) venous phase showing lesion size (45 × 40 mm).

Alpha-fetoprotein (AFP) was 7.02 UI/mL; liver function was preserved (Child–Pugh class A5), and kidney function was within normal limits (Table 1). A liver biopsy was not pursued, as the diagnosis of HCC was established non-invasively according to current EASL/AASLD guidelines in a cirrhotic liver with LI-RADS 5 imaging features. A CT-guided percutaneous biopsy of the renal lesion performed on July 24, 2024, confirmed ccRCC, with positive immunohistochemical staining for CD10 and PAX8, and negative for CK7. Based on the International Metastatic RCC Database Consortium (IMDC) risk model, the patient was classified as intermediate-risk. IMDC classification was based on standard clinical and laboratory parameters, including hemoglobin level, neutrophil and platelet counts, corrected calcium, and performance status.

Baseline staging also revealed multiple pulmonary nodules, including a 16-mm right perihilar lesion, raising concern for possible metastatic or indeterminate systemic involvement. In addition, several enlarged distant lymph nodes of uncertain attribution were observed, precluding a clear organ-specific staging at presentation. A tissue biopsy of the pulmonary nodules was not pursued due to their small size and central/perihilar location, which posed procedural risk and was not expected to alter initial systemic management.

## 3 Therapeutic Intervention

Following multidisciplinary tumor board discussion, a unified systemic immunotherapy strategy was selected in light of synchronous primary malignancies, patient age, and comorbidities, as well as the availability of a single regimen active against both tumor types. Formal TNM and BCLC staging were not definitively assigned at presentation due to indeterminate pulmonary and nodal findings, and therapeutic planning relied primarily on multidisciplinary clinical assessment.

Given the clinically complex presentation and the suspicion of systemic involvement, systemic treatment strategy with ICIs was initiated on August 28, 2024, following the CheckMate-214 protocol. The patient received nivolumab (3 mg/kg) and ipilimumab (1 mg/kg) IV every 3 weeks for 4 cycles, followed by nivolumab monotherapy (240 mg every 2 weeks, later escalated to 480 mg monthly).

**Table 1:** Baseline clinical and tumor characteristics of the patient.

Parameter	Value
Age, years	79
Sex	Female
ECOG Performance Status	1
Comorbidities	Hypertension (controlled), type 2 diabetes mellitus; history of self-limited atrial fibrillation episodes
Liver Function	Child–Pugh A5
AFP	7.02 UI/mL
Renal Function	Creatinine: 0.8 mg/dL/eGFR: 64 mL/min
HCV Status	HCV-RNA negative (June 2024)
Hepatic Lesion Size (Baseline)	53 × 44 mm
Renal Lesion Size (Baseline)	45 × 40 mm
Pulmonary Nodules	Present; largest 16 mm (right perihilar)
Distant Lymph Nodes	Enlarged, indeterminate attribution
IMDC Risk (RCC)	Intermediate
Portal Vein Thrombosis	Absent

Abbreviations: ECOG, Eastern Cooperative Oncology Group; AFP, alpha-fetoprotein; HCV, hepatitis C virus; IMDC, International Metastatic Renal Cell Carcinoma Database Consortium; RCC, renal cell carcinoma.

## 4 Safety

Treatment was well tolerated, with only grade 1 diarrhea during induction. Immune-related toxicity monitoring included serial complete blood counts, liver and renal function tests, and thyroid function assessments at regular intervals; no clinically significant laboratory abnormalities or endocrine toxicities were observed (Table 2). The patient maintained a good performance status throughout treatment, with no reported deterioration in quality of life.

The nivolumab 3 mg/kg plus ipilimumab 1 mg/kg induction regimen was selected to balance efficacy and tolerability in an elderly patient with multiple comorbidities, following the CheckMate-214 schedule. This dosing was intended to maintain systemic activity across both RCC and HCC while minimizing toxicity risk in the context of cirrhosis and clinical frailty.

**Table 2:** Laboratory monitoring and immune-related adverse events during immunotherapy.

Timepoint	AST	ALT	Total Bilirubin	TSH	Creatinine	Immune-Related AEs (CTCAE)
Baseline	WNL	WNL	WNL	WNL	WNL	None
Month 3	WNL	WNL	WNL	WNL	WNL	Grade 1 diarrhea
Month 6	WNL	WNL	WNL	WNL	WNL	None
Last Follow-up (Feb 2026)	WNL	WNL	WNL	WNL	WNL	None

WNL: Within normal limits (“no clinically significant laboratory abnormalities”).

## 5 Response Assessment

Tumor response was assessed on CT using RECIST v1.1 for the renal lesion. For the hepatic lesion, both RECIST v1.1 and immune-related response criteria (including iRECIST considerations) were applied; the initial increase in size with necrotic changes was interpreted as possible pseudoprogression based on subsequent radiologic evolution and clinical stability.

Overall, the renal lesion showed a reduction in maximum diameter from 45 mm at baseline to 27 mm at best response (approximately −40%), while the hepatic lesion decreased from 53 mm to 30 mm (approximately −43%) after an initial increase consistent with pseudoprogression. Specifically, the renal lesion demonstrated an early reduction of approximately 35% after induction therapy, followed by sustained shrinkage of about 40% at best response, whereas the hepatic lesion, after an initial increase, showed a subsequent reduction of approximately 55% at 9-month follow-up.

At the latest follow-up (February 2026), both lesions remained stable (hepatic lesion approximately 30 mm; renal lesion approximately 27 mm), confirming a durable response.

The total duration of treatment and follow-up extended from August 2024 (treatment initiation) to February 2026 (latest follow-up), corresponding to approximately 18 months. The patient remains on treatment.

## 6 Clinical Course

Restaging CT on December 23, 2024, showed a partial response (PR) in the renal tumor (29 × 27 mm vs. 45 × 40 mm baseline), while the hepatic lesion appeared to increase in size (68 × 73 mm vs. 53 × 44 mm) with signs of necrosis and peripheral capsule—interpreted as pseudoprogression (Fig. 2A–F).

**Figure 2:**
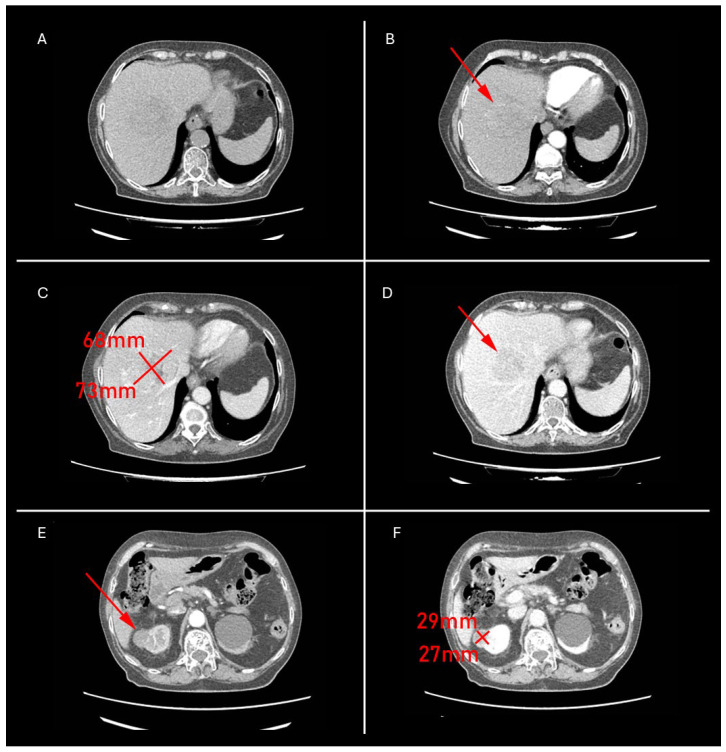
CT scan at 5-month follow-up. (**A**–**D**) Hepatic lesion (HCC): (**A**) non-contrast phase; (**B**) arterial phase showing persistent arterial hyperenhancement (red arrow); (**C**) venous phase demonstrating increased lesion size (68 × 73 mm); (**D**) delayed phase showing central hypodensity and reduced contrast enhancement consistent with intratumoral necrosis and pseudoprogression (red arrow). (**E**,**F**) Renal lesion (RCC): (**E**) arterial phase showing partial response (red arrow); (**F**) venous phase showing significant size reduction (29 × 27 mm vs. 45 × 40 mm).

A follow-up CT scan in May 2025 showed marked shrinkage of the hepatic lesion (30 × 23 mm), consistent with delayed PR, and a stable renal lesion (27 × 25 mm), confirming a durable dual tumor response to treatment (Fig. 3A–F).

**Figure 3:**
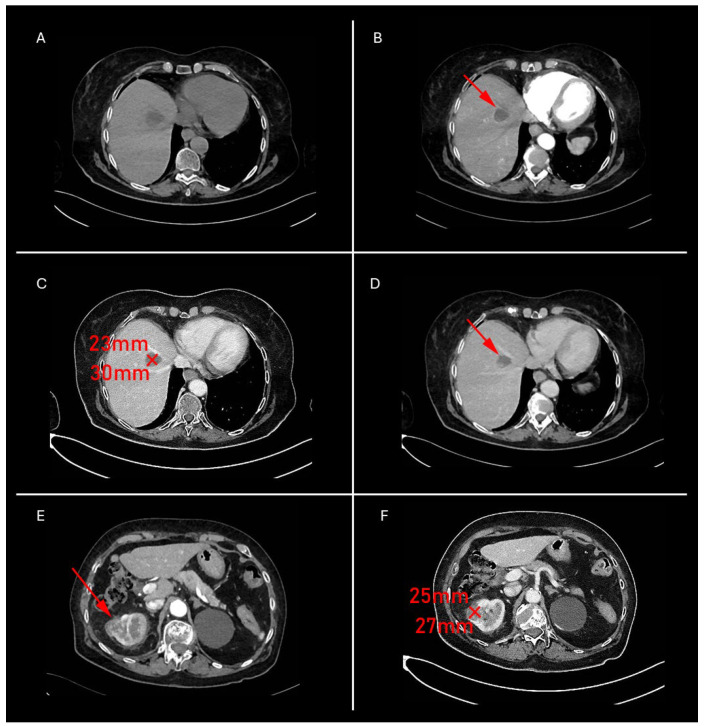
CT scan at 10-month follow-up. (**A**–**D**) Hepatic lesion (HCC): (**A**) non-contrast phase; (**B**) arterial phase showing residual arterial hyperenhancement (red arrow); (**C**) venous phase demonstrating significant reduction in lesion size (30 × 23 mm); (**D**) delayed phase confirming treatment response following prior pseudoprogression (red arrow). (**E**,**F**) Renal lesion (RCC): (**E**) arterial phase showing further regression of the lesion (red arrow); (**F**) venous phase showing sustained size reduction (27 × 25 mm), consistent with a durable therapeutic response.

No new lesions were identified, and the largest pulmonary nodule (16 mm at baseline, right perihilar) remained stable at subsequent evaluations, including December 2024 and May 2025, with no new pulmonary lesions detected. Another CT scan performed in December 2025 showed findings comparable to those of May 2025, confirming sustained disease control. At the latest follow-up in February 2026, clinical and radiologic assessment confirmed stable disease, with lesion measurements reported as substantially unchanged compared to the previous imaging. The patient maintained a good performance status (ECOG 1) and is continuing immunotherapy without complications (Fig. 4). At the time of writing (April 2026), the patient remains on treatment, with an overall treatment duration of approximately 20 months.

Alpha-fetoprotein levels remained within normal limits throughout follow-up (baseline 7 UI/mL and consistently <7 UI/mL thereafter).

**Figure 4:**
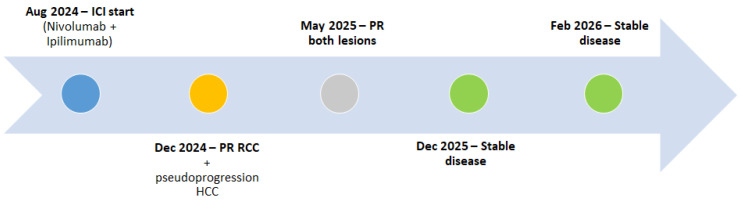
Treatment timeline. ICI starts in Aug 2024, early PR RCC with pseudoprogression HCC in Dec 2024, PR both lesions in May 2025, stable disease in February 2026.

## 7 Discussion

Checkpoint inhibitors have significantly transformed the therapeutic landscape of several solid tumors, including RCC and HCC, both of which demonstrate immunogenic potential. In metastatic RCC, the combination of nivolumab and ipilimumab is a first-line standard for patients with intermediate- or poor-risk disease, based on the pivotal CheckMate-214 trial [1].

In HCC, immunotherapy has also gained momentum and ICI–based combinations have significantly expanded treatment options in advanced disease [2]. The phase I/II CheckMate-040 study first demonstrated that dual immune checkpoint inhibition with nivolumab and ipilimumab could induce durable responses in patients with advanced HCC, leading to accelerated approval in second-line settings [3]. More recently, the phase III CheckMate-9DW trial evaluated the same combination (NIVO + IPI) versus standard tyrosine kinase inhibitors (sorafenib or lenvatinib) in the first-line setting for advanced HCC. Preliminary data presented in 2024 indicated a trend toward improved overall survival, suggesting that dual checkpoint blockade may offer a viable alternative to current anti-PD-L1/VEGF combinations, particularly in selected patients with favorable immune profiles and preserved liver function [4].

While current first-line therapy for HCC is dominated by the combination of atezolizumab and bevacizumab, our case illustrates the clinical plausibility of nivolumab and ipilimumab in complex, real-world settings—especially when systemic coverage for multiple immunogenic tumors is desired [5,6]. This case is unique in that both the HCC and RCC lesions responded to the same immunotherapy regimen, despite their differing tumor biology and anatomical origins.

### 7.1 Immunologic Landscape of RCC and HCC

RCC and HCC, though arising in distinct organs and microenvironments, share several immunological characteristics that make them susceptible to checkpoint blockade. Both tumors often express PD-L1 and harbor tumor-infiltrating lymphocytes (TILs), particularly CD8^+^ T cells [7,8]. RCC is known for its high neoantigen burden and immune infiltrate, while HCC presents more heterogeneously, depending on etiology, underlying liver disease, and inflammation. Interestingly, in patients with cured hepatitis C virus (HCV), like our patient, the immunosuppressive hepatic environment may be partially reversed, allowing for improved ICI responsiveness [9].

Our patient also presented with favorable clinical biomarkers: preserved liver function (Child–Pugh A5) and normal AFP levels. These features are associated with improved outcomes under ICI-based therapies and were consistent with the patient’s observed delayed but significant radiologic response in the liver [10].

### 7.2 Pseudoprogression and Immune-Related Radiologic Patterns

One of the most notable findings in this case was the differential temporal response between the two malignancies. The ccRCC responded rapidly and predictably, while the HCC lesion initially appeared to enlarge before showing a marked reduction at subsequent follow-up. This delayed response is consistent with immune-related pseudoprogression, a phenomenon well-documented in ICI-treated malignancies, including HCC [11,12].

In HCC, pseudoprogression may result from immune cell infiltration, hemorrhage, or necrosis, mimicking tumor growth on imaging. In our case, the hepatic lesion demonstrated peripheral colliquation and the development of a pseudocapsule, suggesting evolving immune activity rather than true progression. This underlines the importance of using immune-specific radiologic criteria—such as iRECIST or mRECIST for HCC—in the assessment of immunotherapy response, especially when RECIST 1.1 may lead to premature discontinuation [13,14].

### 7.3 Synchronous Dual Primary Tumors and ICI: A Rare Clinical Situation

The coexistence of synchronous primary malignancies poses significant therapeutic challenges, particularly when both tumors are advanced or metastatic. There is limited clinical guidance for such scenarios, and few case reports document systemic therapies capable of targeting both tumors simultaneously. Although reports of synchronous dual primary tumors treated with ICIs remain limited, emerging case-based evidence across different solid malignancies [15,16] suggests that unified systemic immunotherapy strategies may be feasible in selected patients.

The rationale for using a single immunotherapy protocol in this case was supported by the shared immunogenicity of RCC and HCC, and by emerging data from trials such as CheckMate-9DW suggesting the viability of IPI–NIVO in HCC as well. Recent expanded analyses from CheckMate-9DW, presented at major international oncology meetings (ASCO and ESMO 2025), suggest that nivolumab plus ipilimumab may provide meaningful response rates and survival outcomes compared with sorafenib or lenvatinib in the first-line treatment of advanced HCC, further supporting the potential activity of this PD-1/CTLA-4 combination [17].

Alternative systemic options were considered for each tumor type. In HCC, first-line standards include atezolizumab–bevacizumab and the STRIDE regimen (durvalumab–tremelimumab), as well as tyrosine kinase inhibitors in selected patients [18]. In metastatic RCC, combinations such as pembrolizumab–axitinib or nivolumab–cabozantinib represent validated approaches [19,20,21]. However, in the context of synchronous immunogenic malignancies, nivolumab plus ipilimumab was favored as a unified strategy capable of providing systemic activity across both tumors while maintaining an acceptable safety profile in an elderly, comorbid patient.

Although cases of dual primary tumors treated with ICIs are rare, a few reports exist. For instance, nivolumab was used successfully in patients with concurrent melanoma and lung cancer, or HCC and urothelial carcinoma [22]. However, to our knowledge, this is the first documented case of combined nivolumab–ipilimumab leading to radiologic benefit in both RCC and HCC. Such findings support further investigation into immune-based strategies that transcend organ-specific histology and exploit shared tumor-immune interactions.

A liver biopsy was not performed for the hepatic lesion because the diagnosis of HCC was established non-invasively according to current international guidelines (EASL and AASLD) [23].

In patients with cirrhosis and typical imaging features, such as arterial phase hyperenhancement and venous washout (LI-RADS 5), as observed in this case, a non-invasive diagnosis of HCC can be established, and biopsy is not required according to current international guidelines. Conversely, a percutaneous biopsy was deemed necessary for the renal mass, which lacked pathognomonic imaging features and required histological confirmation to guide treatment. As the selected therapeutic strategy involved systemic immunotherapy active against both HCC and RCC, a liver biopsy was not expected to alter clinical management.

Although a liver biopsy might have definitively excluded the possibility of a solitary metastasis from renal RCC, several factors make this scenario unlikely: (1) the radiologic features were characteristic of HCC and not suggestive of metastatic disease; (2) no additional distant metastases were identified on staging CT; and (3) the hepatic lesion demonstrated a delayed but favorable response pattern commonly observed with immunotherapy in HCC.

Taken together, these findings provide strong clinical and radiologic support for the diagnosis of synchronous primary HCC rather than metastatic RCC.

This approach balanced diagnostic accuracy with procedural prudence in a frail, elderly patient with multiple comorbidities.

### 7.4 Elderly Patients and Comorbidities: Still Suitable for Dual ICI?

Another important element of this case is the patient’s age and comorbidities (diabetes, hypertension, and prior HCV infection). Elderly patients are often excluded from clinical trials due to concerns about tolerability, particularly with dual checkpoint blockade. However, recent analyses suggest that older adults may derive similar or even greater benefit from ICI therapy compared to younger cohorts, possibly due to age-related shifts in immune surveillance and T-cell exhaustion [24,25,26].

In our case, the patient tolerated nivolumab plus ipilimumab exceptionally well, with only transient grade 1 diarrhea during induction. There were no hepatic immune-related adverse events despite her prior HCV infection, aligning with data suggesting acceptable safety of ICIs in patients with resolved viral hepatitis [27]. These findings support the broader applicability of immunotherapy even in older or frail populations, particularly when the anticipated therapeutic benefit is high.

### 7.5 Limitations and Clinical Implications

The primary limitation of this report is its single-patient nature, which precludes generalizability. Additionally, the diagnosis of HCC was radiologic, albeit supported by LI-RADS 5 classification and MRI features highly specific for HCC in cirrhotic and post-HCV livers. Furthermore, biomarker testing such as PD-L1 expression, microsatellite instability (MSI), or tumor mutational burden (TMB) was not available, representing a limitation in treatment stratification. This report is limited by its single-patient nature and the lack of biomarker-driven immunotherapy selection; therefore, its clinical implications should be interpreted as hypothesis-generating rather than practice-changing. Nevertheless, the dual response and well-tolerated regimen offer valuable clinical insight. Our experience suggests that in cases of multiple synchronous tumors with immunologic potential, a unified systemic immunotherapy approach can be both feasible and effective. As immunotherapy expands across indications, real-world cases like this can inform clinical decision-making in the absence of formal trial data and highlight areas for further translational research [28,29,30].

## 8 Conclusion

This case illustrates the potential efficacy and safety of dual ICB with nivolumab and ipilimumab in the management of synchronous immunogenic malignancies such as hepatocellular carcinoma and renal cell carcinoma. Despite the distinct biological and anatomical characteristics of these tumors, both lesions responded to a unified immunotherapeutic strategy, achieving durable radiologic responses for approximately 18 months with ongoing treatment as of April 2026 and minimal toxicity in an elderly patient with multiple comorbidities.

Beyond the individual clinical outcome, this report highlights the feasibility of adopting a single systemic immunotherapy strategy in complex clinical scenarios involving synchronous primary malignancies, particularly when tumors share immunogenic features. The case also emphasizes the importance of careful radiologic interpretation during immunotherapy, especially in hepatocellular carcinoma, where atypical response patterns such as pseudoprogression may occur and may otherwise lead to premature treatment discontinuation.

Although based on a single case, these findings contribute to the limited body of literature describing synchronous solid tumors successfully managed with ICIs. Our experience supports the potential role of immune-based strategies capable of targeting multiple malignancies simultaneously and underscores the need for further studies exploring shared immunologic mechanisms and optimal therapeutic approaches for patients presenting with concurrent cancers.

## Data Availability

The authors confirm that the data supporting the findings of this study are available within the article.

## Supplementary Materials

The supplementary material is available online at https://www.techscience.com/doi/10.32604/or.2026.078258/s1.
