# Supplementary material for: Dual Immune Checkpoint Blockade in Synchronous Hepatocellular and Renal Cell Carcinoma: A Real-World Case Report of Durable Dual Response
Source: Oncol Res. 2026 Jun 16;34(7):30. doi: 10.32604/or.2026.078258 (PMC13292052; doi:10.32604/or.2026.078258)
Supplement: Supplementary file 1 [file OncolRes-34-78258-s001.zip › Supplementary_Material_S1.docx]

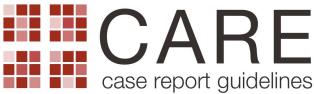
CARE Checklist of information to include when writing a case report
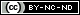


**Topic Item Checklist item description Reported on Line**

**Title 1** The diagnosis or intervention of primary focus followed by the words “case report” :

**Title identifies the article as a case report Page 1**

**Key Words 2** 2 to 5 key words that identify diagnoses or interventions in this case report, including "case report": **Page 2**

**Abstract**

**(no references)**

**3a** Introduction: What is unique about this case and what does it add to the scientific literature?: **Page 1- 2 (Abstract-Conclusions)**

**3b** Main symptoms and/or important clinical findings : **Page 3 (Case history)**

**3c** The main diagnoses, therapeutic interventions, and outcome: **Page 1–2 (Abstract)**

**3d** Conclusion—What is the main “take-away” lesson(s) from this case? **Page 1–2 (Abstract – Conclusions)**

**Introduction 4** One or two paragraphs summarizing why this case is unique (**may include** reference**s**) **Page 2 (Introduction)**

**Patient Information 5a** De-identified patient specific information: **Page 3–4 (Case history)**

**5b** Primary concerns and symptoms of the patient **Page 3–4 (Case history)**

**5c** Medical, family, and psycho-social history including relevant genetic information: **Page 3–4 (Case history)**

**5d** Relevant past interventions with outcomes: **Page 3–4 (Case history)**

**Clinical Findings**

**Timeline**

**Diagnostic Assessment**

**Therapeutic Intervention**

**Follow-up and Outcomes**

1. Describe significant physical examination (PE) and important clinical findings **Page 3–4** **(Case history / Clinical findings)**
2. Historical and current information from this episode of care organized as a timeline **Page 5–6 (Case history/Clinical course)**

**8a** Diagnostic testing (such as PE, laboratory testing, imaging, surveys). **Page 3–4 (Diagnostic assessment/ Case history)**

**8b** Diagnostic challenges (such as access to testing, financial, or cultural **N/A**

**8c** Diagnosis (including other diagnoses considered) **Page 8–11 (Discussion – Diagnostic reasoning)**

**8d** Prognosis (such as staging in oncology) where applicable **Page 3-6 (Case history / Clinical course)**

**9a** Types of therapeutic intervention (such as pharmacologic, surgical, preventive, self-care) **Page 3 – 4 (Case history /Therapeutic intervention)**

**9b** Administration of therapeutic intervention (such as dosage, strength, duration) **Page 3–4 (Case history/Therapeutic intervention)**

**9c** Changes in therapeutic intervention (with rationale) **Page 3–4 (Case history/Therapeutic intervention)**

**10a** Clinician and patient-assessed outcomes (if available) **Page 5–6 (Clinical course; Safety)**

**10b** Important follow-up diagnostic and other test results **Page 5–6 (Clinical course; Safety)**

**10c** Intervention adherence and tolerability (How was this assessed?) **Page 5-6 (Clinical course; Safety)**

**10d** Adverse and unanticipated events **Page 5–6 (Clinical course; Safety)**

**Discussion 11a** A scientific discussion of the strengths AND limitations associated with this case report

**Page 8–11 (Discussion – Limitations and clinical implications)**

**11b** Discussion of the relevant medical literature **with references Page 8 –11 (Discussion)**

**11c** The scientific rationale for any conclusions (including assessment of possible causes) **Page 8–11 (Discussion – Scientific rationale)**

**11d** The primary “take-away” lessons of this case report (without references) in a one paragraph conclusion **Page 11 (Conclusion)**

**Patient Perspective 12** The patient should share their perspective in one to two paragraphs on the treatment(s) they received **Page 4–5 (Case history / Safety)**

**Informed Consent 13** Did the patient give informed consent? Please provide if requested **Yes X** **No
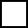
**
